# Bilateral hip stability variation in the functional ambulation and kinetic parameters after total hip arthroplasty during leveled walking

**DOI:** 10.3389/fbioe.2024.1415645

**Published:** 2024-08-14

**Authors:** Amany E. Abd-Eltawab, Dalia Mahmoud Abdelmonem Elsherbini, Eman Mohamad El Nashar, Shaker Hassan Alshehri, Ali Alhefzi, Mamdouh Eldesoqui, Mohamed El-Sherbiny

**Affiliations:** ^1^ Physical Therapy and Health Rehabilitation Department, College of Applied Medical Sciences, Jouf University, Sakaka, Saudi Arabia; ^2^ Biomechanics Department, Faculty of Physical Therapy, Cairo University, Giza, Egypt; ^3^ Department of Clinical Laboratory Sciences, College of Applied Medical Sciences, Jouf University, Sakaka, Saudi Arabia; ^4^ Department of Anatomy, College Medicine, King Khalid University, Abha, Saudi Arabia; ^5^ Department of Orthopedic, College of Medicine, King Khalid University, Abha, Saudi Arabia; ^6^ Department of Basic Medical Sciences, College of Medicine, AlMaarefa University, Riyadh, Saudi Arabia; ^7^ Department of Human Anatomy and Embryology, Faculty of Medicine, Mansoura University, Mansoura, Egypt

**Keywords:** hip arthroplasty, walking stability, ambulation parameters, ground reaction force, gender variation

## Abstract

**Objectives:**

This study determines gender variation, comparing the significance level between men and women related to functional ambulation characteristics after hip arthroplasty. The study focuses on the broader female pelvis and how it affects the rehabilitation regimen following total hip arthroplasty.

**Materials and Methods:**

In this cross-sectional study, 20 cases of right hip arthroplasty were divided into 10 male and 10 female cases, aged 40–65 years. The functional ambulation parameters (walking cadence, gait speed, stride length, and gait cycle time) were acquired from the GAITRite device, as well as kinematic values for hip frontal plane displacement and kinetic parameters for ground response force in the medial–lateral direction.

**Results:**

An independent *t*-test showed a significant difference in the kinematic parameter variables for the anterior superior iliac spine, more significant trochanter displacement, and hip abduction angle between the operated and non-operated limbs for each group separately. Regarding the functional ambulation parameters, there was a significant difference in the walking cadence between the operated and non-operated limbs of both male and female groups. Moreover, the output variables of ground reaction force measures revealed significant differences between their operated and non-operated limbs. The linear regression model used was consistent with the current results, demonstrating a weak negative correlation between the abduction angle of the operated hip and gait speed for both male and female groups.

**Conclusion:**

Based on the findings, we draw the conclusion that improving a rehabilitated physical therapy program for the abductors of both male and female patients’ operated and non-operated limbs is essential for normalizing the ground reaction force value, avoiding focus on the operated hip, and reducing the amount of time that the operated hip’s abductors must perform. This involves exposing the surgically repaired limb to the risk of post-operative displacement or dislocation, particularly in female patients.

## 1 Introduction

Total hip arthroplasties (THAs) are safe and effective surgical interventions for relieving pain and improving physical function caused by arthritis or other reasons for hip deformation and pain. More than one million THAs are annually performed worldwide (Kiernan, 2020; Stolarczyk et al., 2022). Significant alterations in static and muscle parameters are present after THA and gait parameters (Martinez et al., 2022). Following a total hip replacement, most patients have mild hip impairments, limiting their ability to do daily tasks, such as stiffness and hip discomfort in the region of the abductors. In addition to hip muscle weakness that results in hip dysfunction, these patients may still experience functional gait abnormalities in their walking cadence and speed, particularly in the first year following surgery (Frost et al., 2006).

Kolářová et al. (2020) examined postural stability and hip joint range of motion 1 year following hip arthroplasty, focusing on the importance of weight-bearing exercise and rehabilitation programs. They observed improvement in the late stance phase of walking. Similarly, Frost et al. (2006) conducted a comparative study to compare the hip peak torque of the unilateral total hip arthroplasty group to the control group. They reported that peak hip flexor torque decreased compared to the control group, demonstrating the value of an exercise program and a home program for improving hip performance in arthroplasty patients.

Approximately 60% of the samples, aged 55–64 years, suffer from arthrosis (Beaulieu et al., 2010). Several studies examined the difference in kinematic parameters between normal hip joints and arthrosis hip joints. In arthrosis hip joints, they reported significant slow gait velocity, shorter step lengths, and longer double-limb stance times. In addition, the range of motion for the hip, ankle, pelvis, and trunk varies in the sagittal, frontal, and transverse planes between the two groups (Zeni et al., 2015; Renkawitz et al., 2016; Constantinou et al., 2017).

Additionally, range of motion, postural hip stability, and hip muscle strength were evaluated between the uninvolved limb (Trudelle-Jackson et al., 2002) and operative hip arthroplasty, demonstrating the significance of postural stability and weight bearing in the postoperative phase. Unlu et al. (2007) compared the efficacy of home exercise programs to in-hospital exercise programs on hip joint muscle strength, gait speed, and cadence for patients with total hip arthroplasty 1 year following surgery. Their findings showed that maximum isometric hip abduction torques improved only in the first and second groups. Meanwhile, gait speed and cadence improved in all three groups.

There is a dearth of information about the effect of sex on ambulation parameters for total hip arthroplasty, except for estimating the magnitude of ground reaction force and its effect on hip stability. Thus, this study compares the gender variation between men and women regarding functional ambulation characteristics after hip arthroplasty. Another aim is to obtain bilateral hip stability for total hip arthroplasty and non-operated one when walking by evaluating the lateral stability of the hip and the pelvic tilt, which are considered important prerequisites in the gait cycle for appropriate walking success. The point of interest in the present research is protecting the operated arthroplasty hip from further related injuries during daily activities. In addition, it gives further prophylactic output measurements for the non-operated hip to prevent feeling overburdened from being concentrated on during walking. From an ergonomic point of view, critical instructions for those with hip arthroplasty are to pay attention when walking upstairs or on inclined ramps, which increases the liability of dislocating the operated hip.

## 2 Materials and Methods

### 2.1 Study design

A cross-sectional investigation was conducted to contrast the operated and non-operated hip arthroplasty joints. The sexual dimorphism concerning the functional ambulation parameters (FAP) for walking cadence, gait speed, stride length, and gait cycle time, in addition to the impact of sex, was evaluated. Furthermore, the kinematic hip angular displacement for the abduction range of motion was identified and contrasted across all gait phases. Kinetic gait analysis also recorded the mean value of the ground response force in the *Y*-direction for both hip joints within the group and between male and female members categorized into two groups.

### 2.2 Participants and power analysis

In this study, 20 patients with right hip arthroplasty were included and divided into two groups: 10 male patients with right hip arthroplasty and 10 female patients with right hip arthroplasty. The cases were chosen from the outpatient clinic of Cairo University’s physical therapy faculty. The selection criteria for the patients were those who had undergone unilateral right hip arthroplasty, aged 40–60 years and were chosen based on the affected right hip, which was degenerative and required a total hip replacement. They participated in this trial for about 1 year after surgery (Mazzoli et al., 2017). Their weight and height matched, ranging from 70 to 85 kg and 1.6–1.7 cm, respectively. Any musculoskeletal deformities, congenital anomalies in the joints of lower extremities, organic or infectious hip diseases, any history of neurologic disorders, or oncologic arthroplasty cases were excluded from this study.

The sample size was determined using G*power software version 3.1.9.7 (Universities, Dusseldorf, Germany), and the size was chosen based on the difference between the two independent group means. Size effect (d) was estimated from the literature between two female and male groups (Queen et al., 2019). The means ± SD for women vs*.* men was (1.13 ± 0.19), (0.82 ± 0.15), (0.92 ± 0.14), and (0.72 ± 0.11) for stride length, cadence, speed, and cycle time, respectively, and (0.0715 ± 0.01 and 0.063 ± 0.01) for medio-lateral ground reaction force. Following these hypotheses, the sample sizes for functional ambulation parameters and ground reaction force were 12, 22, and 36. The power (80%) was acquired to determine the influence of these sizes at the alpha level of 5%, using the smallest size.

### 2.3 Instrumentations

The current study focuses mostly on two systems.

#### 2.3.1 The GAITRite system

One component of the GAITRite system is a movable walkway with sensors that sense pressure. The walkway keeps track of the sensors’ distance apart and activity state and then it inputs these data into the application software to compute temporal and spatial gait metrics for every step. Gait patterns were evaluated using a pressure-sensitive walkway system (GAITRite-System, GS), and Motognosis Labs Software with a Microsoft Kinect Sensor (MKS) was used to verify the device’s accuracy and durability (Webster et al., 2005). Walking cadence, gait speed, stride length, and gait cycle time were determined and compared between two groups using spatiotemporal characteristics derived from this device.

#### 2.3.2 Qualysis motion capture system

A force plate component was combined with a Qualysis motion capture system to extract the kinetic data for this investigation. The system construction, which includes six high-velocity infrared Pro-Reflex cameras and an AMTI (Advanced Mechanical Technology Inc., United States) force plate, is mounted in the center of a walkway. To investigate a human body’s force on a plate in such a setup is necessary, and a cable is used to link the force plate to a computer unit. An internal amplifier amplifies the signals from the plate before feeding them into an analogue to a digital (A/D) converter. Therefore, the digitization voltage values represent the system’s final output. The AMTI force plate employs a coordinate-oriented system.

### 2.4 Procedure

The mean values of walking cadence, gait speed, stride length, and gait cycle time showed a significant difference, as indicated by spatiotemporal parameters collected from the GAITRite device. To ensure that the walking pattern is normal and to identify any significant differences in the spatiotemporal gait parameters for the selected cases, each patient was asked to walk as normally as possible without using a gait training device. This required each patient to walk barefoot along the GAITRite device’s walkway three times. The mean values of walking cadence, gait speed, stride length, and gait cycle time were significantly recorded, as evidenced by spatiotemporal parameters gathered by the GAITRite device.

In addition to documenting and comparing the angular displacement of hip abduction, the anterior superior iliac spine (ASIS), and greater trochanter displacements for the operated and non-operated limbs of both men and women, the Qualisys Motion Capture System was utilized in this study in conjunction with a force plate component to extract the kinetic data. The ground reaction force value in the *Y* direction (ground reaction force, GRF–Y) represented these kinetic data. The double-sided adhesive tape was used to fix the markers at the particular location of the site: 1. the 12th thoracic vertebrae, 2. shoulders to the right and left, 3. sacrum, 4. greater trochanters on the right and left, 5. superior iliac spines on the right and left, 6. suprapatellar regions on the right and left, 7. the tibia’s left and right tuberosities, and 8. knee joints on the right and left, and 9. Toes between the second and third metatarsals. The right and left heels and the ankles’ joints represented the 10th and 11th markers. In order to allow each of the six cameras to identify the locations of the reflecting markers in the walkway’s path field, this system was modified prior to detecting the characteristics of walking. The force plate that settled in the center of the walkway enabled participants to walk over barefoot. They were informed not to target it and to walk normally as much as possible (Senior, 2004).

### 2.5 Statistical analysis

The obtained data were analyzed using GraphPad Prism version 9, which reported the results as the mean ± standard error of the mean (SEM). The Student’s t-test was used to identify the disparities in demographic features between the male and female groups. The effect size between groups was computed using partial eta square. Significance was determined based on a *p*-value of less than 0.05. This study analyzed the association between hip abduction angle and gait speed in the operated limb of both men and women, using a linear regression model with a generalized estimating equations (GEE) correction. Prior to using the parametric assumption, the normality and homogeneity of the variance were statistically analyzed.

## 3 Results

Demographic data were collected for the selected cases, as shown in Table 1. The sample included 20 patients (10 male and 10 female) with no significant difference in the mean value in their ages and body mass indexes (*p* = 0.54 and 0.42), respectively.

**TABLE 1:** Demographic characteristics of study groups.

Variables	Male (*N* = 10)	Female (*N* = 10)	*p*-value between groups
	Mean ± SEM		
Age (years)	47.60 ± 2.90	50.50 ± 3.60	0.54 (ns)
BMI (kg/m^2^)	28.67 ± 0.96	30.23 ± 1.64	0.42 (ns)

− BMI, body mass index, *N*, number.

− Mean ± SEM (standard error of means) is used to provide quantitative data, and an independent Student’s t-test was used.

− (ns), non-significant.

According to the ambulation parameter results (Table 2), a significant difference was seen in the walking cadence between the operated and non-operated limbs within each group (*p* = 0.03). However, no significant differences among the ambulation parameters were found between groups.

**TABLE 2:** Ambulation parameters in the study groups.

Variable	Male				Female				*P* ^*b)*^ between groups	Effect size (η^2^)
	Op	Non-op	MD (95%CI)	*P* ^*a)*^ within group	Op	Non-op	MD (95%CI)	*P* ^*a)*^ within group		
Stride length (m)	0.95 ± 0.15	0.93 ± 0.08	−0.02 (−0.37–0.33)	0.90	062 ± 0.14	0.69 ± 0.14	0.07 (−0.35–0.49)	0.73	0.12	0.13
Walking cadence (steps/min)	88.60 ± 3.19	108.0 ± 8.23	19.4 (2.76–36.04)	0.03*	98.30 ± 9.54	74.80 ± 2.20	−23.50 (−44.07 to −2.93)	0.03*	0.35	0.05
Gait speed (m/s)	0.80 ± 0.05	0.81 ± 0.05	0.01 (0.08–0.10)	0.80	0.52 ± 0.04	0.62 ± 0.07	0.10 (−0.07–0.27)	0.24	<0.001	0.50
Gait cycle time (sec)	1.10 ± 0.16	1.17 ± 0.21	0.07 (−0.39–0.53)	0.74	1.10 ± 0.18	0.84 ± 0.19	−0.26 (−0.81–0.29)	0.33	>0.99	0.00

− Values are shown as mean ± standard error of mean (SEM).

− Op, operated limb; Non-op: non-operated limb; MD: mean difference; CI: confidence interval.

− *P* ^a)^-value within group from independent Samples *t*-test.

− *P* ^*b)*^-value between men and women for operated limb from independent Samples *t*-test.

− η^2^, partial eta square between men and women for the operated limb.

**FIGURE 1:**
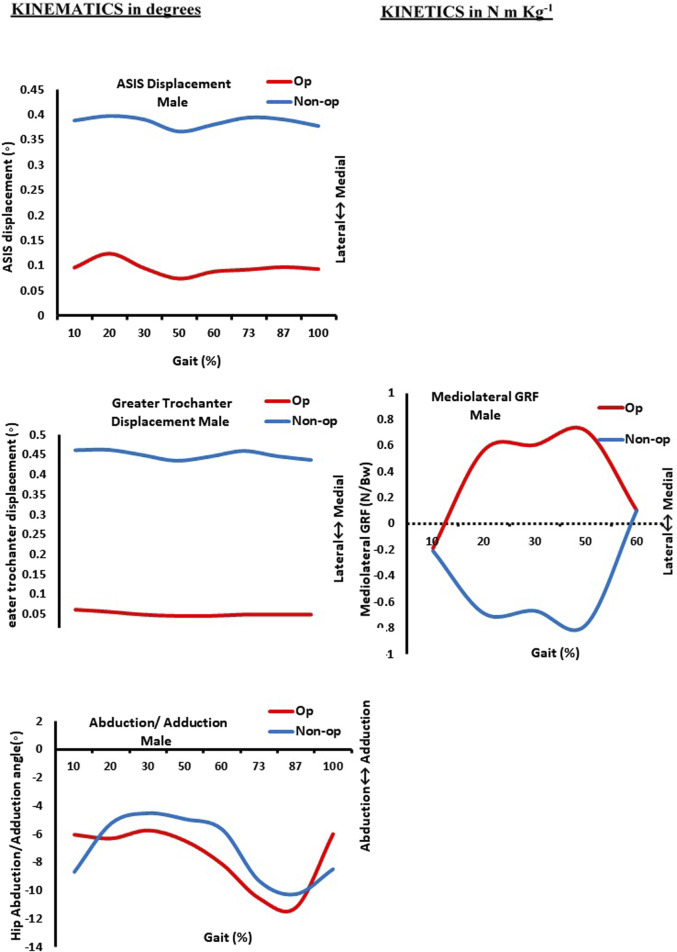
Kinematics (in degrees) and kinetics (in N m/kg) curves as function of normalized gait (in %) in men. Op: operated limb; Non-op: non-operated limb; ASIS: anterior superior iliac spine. 10% (initial contact); 20% (loading response); 30% (mid-stance); 50% (terminal stance); 60% (pre-swing); 73% (initial swing); 87% (mid-swing); and 100% (terminal swing).

**FIGURE 2:**
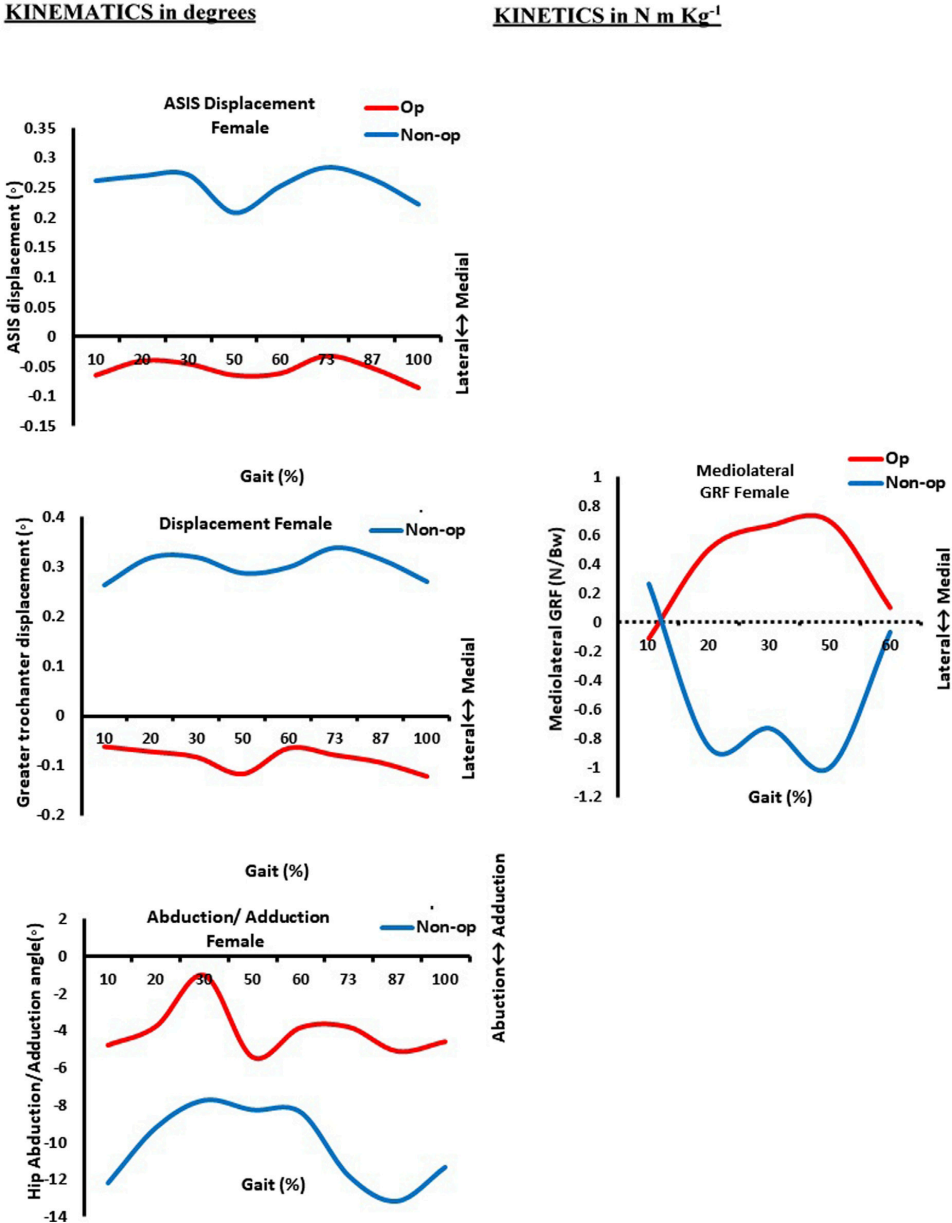
Kinematics (in degrees) and kinetics (in N m/kg) curves as a function of normalized gait (in %) in women. Op: operated limb; Non-op: non-operated limb; ASIS: anterior superior iliac spine. 10% (initial contact); 20% (loading response); 30% (mid-stance); 50% (terminal stance); 60% (pre-swing); 73% (initial swing); 87% (mid-swing); and 100% (terminal swing).

**FIGURE 3:**
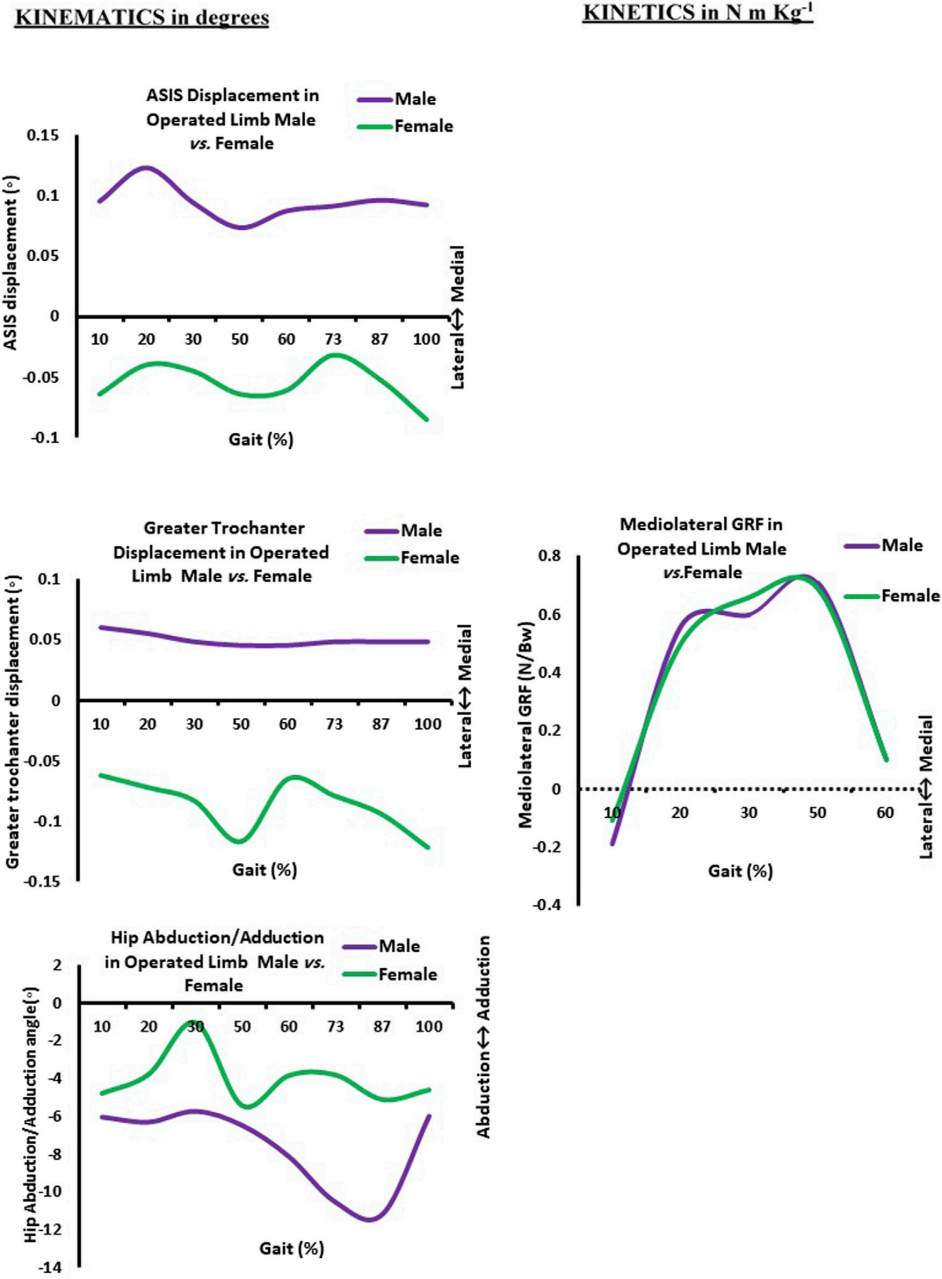
Kinematics (in degrees) and kinetics (in N m/kg) curves as a function of normalized gait (in %) in the operated limb in male *vs.* female. ASIS: anterior superior iliac spine. 10% (initial contact); 20% (loading response); 30% (mid-stance); 50% (terminal stance); 60% (pre-swing); 73% (initial swing); 87% (mid-swing); and 100% (terminal swing).

Table 3 and Figures 1–3 present the kinetics and kinematic parameter differences for the study groups. It showed significant differences (*p* = 0.006) in the anterior superior iliac spine (ASIS) displacement for the male group between the operated and non-operated limbs. As both the ASIS for operated and non-operated limbs were directed medially, the non-operated one shifted more medially (Figure 1). A highly significant difference (*p* > 0.001) for the female group was approved. The ASIS of the non-operated limb of the female group was directed more medially compared with the ASIS of the operated limb that was directed laterally (Figure 2).

**TABLE 3:** Kinematics and kinetics parameters in the study groups.

Variable	Male				Female				*P* ^*b)*^ between groups	Effect size (η2)
	Op	Non-op	MD (95%CI)	*P* ^*a)*^ within group	Op	Non-op	MD (95%CI)	*P* ^*a)*^ within group		
Kinematics										
ASIS displacement (◦)	0.09 ± 0.06	0.39 ± 0.07	0.29 (0.10–0.49)	0.006**	−0.06 ± 0.06	0.25 ± 0.05	0.31 (0.15–0.47)	<0.001***	0.09	0.15
Greater trochanter displacement (◦)	0.05 ± 0.03	0.45 ± 0.06	0.40 (0.21–0.59)	<0.001***	−0.09 ± 0.05	0.30 ± 0.04	0.39 (0.25–0.53)	<0.001***	0.11	0.14
Hip abduction (◦)	−7.57 ± 1.11	−7.17 ± 0.81	0.39 (−2.49–3.28)	0.78	−4.01 ± 1.70	−10.22 ± 1.22	−6.20)-10.60 to-1.81)	0.008**	0.10	0.15
Kinetics mediolateral GRF (N/BW)										
Initial contact phase	−0.19 ± 0.04	−0.21 ± 0.04	−0.03 (−0.15–0.59)	0.65	−0.11 ± 0.02	0.26 ± 0.05	0.38 (0.27–0.48)	<0.001***	0.11	0.14
Loading response phase	0.56 ± 0.04	−0.69 ± 0.05	−1.25 (−1.39 to −1.11)	<0.001***	0.50 ± 0.05	−0.86 ± 0.07	−1.36 (-1.55 to-1.18)	<0.001***	0.37	0.05
Mid-stance phase	0.60 ± 0.06	−0.67 ± 0.07	−1.27 (−1.46 to −1.08)	<0.001***	0.66 ± 0.05	−0.78 ± 0.09	−1.44 (-1.65 to-1.23)	<0.001***	0.41	0.04
Terminal-stance phase	0.71 ± 0.07	−0.78 ± 0.08	−1.49 (−1.70 to −1.28)	<0.001***	0.69 ± 0.07	−1.00 ± 0.09	−1.69 (−1.94 to −1.44)	<0.001***	0.82	0.00
Pre-swing phase	0.10 ± 0.01	0.10 ± 0.03	−0.00 (−0.07 to –0.07)	0.97	0.10 ± 0.02	−0.07 ± 0.01	−0.16 (−0.21 to −0.12)	<0.001***	0.80	0.00

− Values are shown as mean ± standard error of mean (SEM).

− Op, operated limb; Non-op, non-operated limb; MD, mean difference; CI, confidence interval; ASIS, anterior superior iliac spine; GRF, ground reaction force.

− *P* ^a)^-value within group from independent samples *t*- test.

− *P* ^*b)*^-value between men and women for operated limb from independent Samples *t*-test.

− η^2^, partial eta square between men and women for the operated limb.

Thus, Figure 3 revealed that the ASIS of the female-operated limb was directed laterally compared to the ASIS of the male-operated limb, which was directed medially. In addition, a highly significant difference (*p* > 0.001) was found in the greater trochanter displacement during walking between operated and non-operated limbs for both male and female groups. As shown in Figure 1, the greater trochanters of both limbs of the male group were directed medially, but the greater trochanter of the operated limb shifted medially compared to the non-operated one. In contrast to Figure 2, the greater trochanter of the non-operated limb of the female group is directed medially compared to laterally shifting one of the operated limbs. As shown in Figure 3, the greater trochanter displacement of the female operated limb is also directed laterally compared to the greater trochanter displacement of the male-operated limb, which is directed medially.

Additionally, the hip abduction angle showed a significant difference (*p* = 0.008) between the non-operated and operated limbs for the female group only. Figure 1 shows both male limbs that were operated on and those that were not. On the other hand, the non-operated female limb in Figure 3 was more abducted than the operated limb pointing in the direction of adduction. As a result, at approximately 30% of the gait cycle, or the mid-stance phase, the abduction hip angle of the female-operated limb decreases in Figure 3. In the meantime, the male leg that was operated on has its hip abduction angle measured. At 87% of the gait cycle, which is the terminal swing phase, it progressively increases.

Regarding the difference in the mean value of medio-lateral GRF for each group during walking, the current results showed that there was a highly significant difference for all five sub-phases of the gait cycle, which are initial contact, loading response, mid-stance, terminal stance, and pre-swing between operated and non-operated limbs of the female group. The GRF is directed medially for an operated limb and laterally for a non-operated limb (Figure 2). Meanwhile, only three sub-phases—loading response, mid-stance, and terminal stance—registered a highly significant difference for the male group during walking (*p* > 0.001) as the GRF is also directed medially for an operated limb and laterally for a non-operated limb (Figure 1). It was approved in Figure 3, where the mean value of the GRF is slightly higher and more medially directed toward the hip of the operated female limb compared to the hip of the same male limb; that was obvious at mid-stance, which represented 30% of the gait cycle during their walking.

**FIGURE 4:**
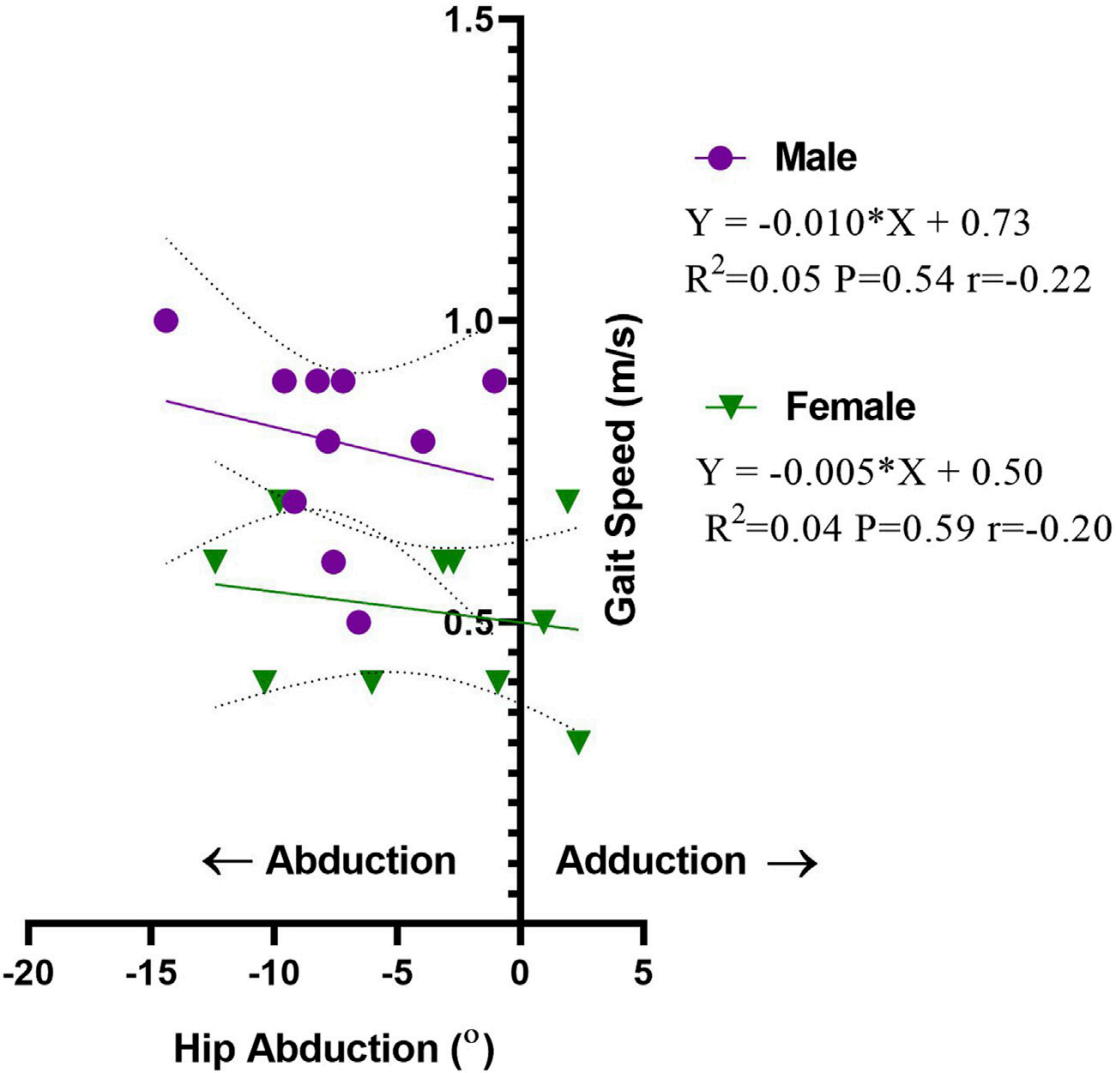
Linear regression of hip abduction angle (◦) *vs.* gait speed (m/s) in the operated limb in men and women.

The linear model regression used in the current study demonstrates a weak negative correlation between hip abduction and gait speed in the operated limb in men and women (r = −0.22, −0.20, respectively). This was statistically insignificant, as indicated by linear regression in men and women. In men, the deviation of the hip abduction angle toward positive adduction explains 5% of the increase in gait speed (*p* = 0.54), in comparison to 4% increase in gait speed in women, (*p* = 0.59) (Figure 4).

## 4 Discussion

In this study, a significant difference in the walking cadence between operated and non-operated limbs within each group was seen. In addition, there was a significant difference in the ASIS displacement for male groups, and a highly significant difference was found in the female group. Moreover, a highly significant difference was found in the greater trochanter displacement within male and female groups. The results also showed a highly significant difference in the mean values of GRF for all sub-phases of the gait cycle for the female group and only three sub-phases for the male group.

These findings are consistent with those of Ornetti et al. (2011), which reported apparent differences in the range of motion of the frontal and sagittal planes around the hip and ankle joints. Furthermore, changes in pelvic and trunk rotations may explain the link between greater trochanter and ASIS displacement. Furthermore, after hip surgery, the range of motion in the non-operated hip increases, which would have been expected in parallel with an increase in the ROM in the operated hip (Leigh et al., 2016). Moreover, Ozan et al. (2014) stated that a mean hip abduction angle is the optimum angle to achieve medial stability of greater trochanter displacement after partial hip arthroplasty. Their study aimed to investigate the effect of greater trochanteric fixation using a multifilament cable to enhance the abductor lever arm in patients with a proximal femoral fracture undergoing partial hip arthroplasty in 12 male and 20 female cases.

In accordance with the results of the current study, Hsu et al. (2007) revealed that the medial reattachment of the greater trochanter improved the abductor mechanism to decrease the rate of hip arthroplasty dislocation, directly eliminating the pain from the femoral-destructed head. Thus, posterior pelvic tilt increases the lateral displacement of the greater trochanter, enhancing the dislocated arthroplasty hip (Choi and Robinovitch, 2018).

Our earlier studies proved that there was a medial displacement in the sacrum of the female group in the *Y*-direction found in all sub-phases of the gait cycle compared to the male group that reflected variation in the value of the medio-lateral GRF between them (Abd-Eltawab et al., 2023). The sacrum, the key to the human skeletal system, links the spine to the iliac bones and remains crucial for hip stability. These sexual differences in the sacrum often determine sex variation in the pelvis and hip regions (Hall, 2019). These findings were compared to the current findings that showed that variations in the iliac bones were linked to variations in the female sacrum. The results of this study indicate that there are notable disparities in ASIS and trochanter displacement between males and females based on variances in the iliac bone.

The current results were consistent with those of Lalevée et al. (2023), which included 31 participants, 68 years of age, who had undergone a minimally invasive hip complete arthroplasty. They evaluated spatiotemporal gait characteristics, electromyography, and vertical ground response force 1 year after surgery in operated hip individuals in an asymptomatic group of the same age and approved decreased sagittal plane moments of the operated hip and pelvic areas. Furthermore, the operated hip’s vertical ground reaction force and spatiotemporal characteristics were lower than in the symptomatic group.

Reductions in the velocity of walking stride length, sagittal hip range of motion, and peak hip abduction were seen when comparing the post-total arthroplasty and control groups were reported by Ewen et al. (2012), which was consistent with our study. In light of the data obtained when comparing total hip arthroplasty groups and resurfacing hip arthroplasty groups, it was discovered that patients with resurfacing hip arthroplasty improved in gait metrics such as walking speed and adduction hip moments compared to another group. Furthermore, the second group demonstrated more prominent abduction hip moments and symmetries in their hip muscle activation postoperatively compared to individuals who underwent total hip arthroplasty (Zhao et al., 2010). Moreover, our earlier studies showed a significant difference in abductor moments between normal female and male cases that were higher in women than men during the gait cycle’s mid-stance and terminal stance phases (Abd-Eltawab et al., 2022).

Sara and Lewis (2023) proved the crucial role of post-operative hip rehabilitation after arthroplasty, where rehabilitation can optimize joint motion, function, and muscle strength. Moreover, the outcomes of gait mechanics can improve after rehabilitation. Moreover, Riley and Killackey (2023) proved that 51% of female and 49% of male patients under 65 years of age improved their pain scores and hip joint function compared to more advanced age after total hip arthroplasty. However, 20%–22% of the selected cases are susceptible to revision and admission within 90 days after operation due to pain and fracture, which negatively affects the hip mechanics while walking.

Based on the current results, measurements of walking speed after 3–5 days post-surgery (baseline) and 1 year of total hip arthroplasty among 207 participants with total hip arthroplasty were studied by Heiberg et al. (2024). A 4-m walking test that was a part of the Short Physical Performance Battery measured walking speed, and the participants were instructed to walk at their comfortable speed on a leveled floor with or without walking aids, and the time was measured in seconds. Walking speed was calculated from the number of seconds taken to walk 4 m, and a decrease in walking speed by 54% after 1 year compared with the baseline was observed. Moreover, Xiong et al. (2024) compared total hip arthroplasty and direct anterior approach related to visual analogue scale (VAS) scores, revealing a significant difference in the mean values of the VAS between both groups, negatively implicating the walking parameters.

Kamimura et al. (2013) studied the correlation between gait speed after total hip arthroplasty and pelvic inclination angle. They collected 36 unilateral female hip arthroplasty patients and evaluated their hip abductors’ muscle strength and the inclination angle of the pelvis and timed go-and-up tests before and after the operation. Their results showed a posterior pelvic inclination affection on the female hip abductor’s strength that decreased their gait speed, functional test, and hip abduction angle after the operation. Thus, the mentioned posterior pelvic tilt in the previous results indicated a change in the greater trochanter and ASIS displacement of the female hip and pelvic region approved in this study that affected female hip abduction angular displacement.

Outcome metrics based on comorbidity profiles, operating time, and length of hospital stay were compared between male and female patients. The findings demonstrated that women had a higher risk of reoperation following total hip arthroplasty; however, men are highly susceptible to reoperation following total knee replacement (Patel et al., 2020). That is because there was a little increase in the mean value of the medio-lateral ground response force in the female group compared to the male group, which may have exposed them to reoperation; these earlier results almost agreed with our results. Solarino et al. (2022) assessed the variations by gender in the total hip arthroplasty outcome and validated the existing findings by demonstrating that in order to enhance their clinical and functional ambulation gait, female individuals require special attention throughout the pre-operative and post-operative phases. Furthermore, Ikutomo et al. (2018) projected that within a year following surgery, the rate of complete hip replacements in female patients would rise because of anomalies in their gait and variations in the strength of their hip abductors muscles when walking.

The current results disagree with those of Queen et al. (2011). They compared the anterolateral approach hip arthroplasty experiences of seven and eight women and stated that the vertical value of the ground response force, the stance time, and the swing time did not differ significantly with time. We measured the GRF in the medio-lateral direction, which may have contributed to the variations between their and current results. In contrast, they measured vertical GRF in the interim. Furthermore, our findings corroborated theirs about the variations in the angular hip displacement in the frontal plane between men and women, explaining the variation in GRF between men and women in the medio-lateral direction.

The limitations of the present study were the small sample size. Effect size assessment revealed that no effect size indices were greater than 0.50 for ambulation, kinetics, and kinematics parameters between men and women for operated limbs. As a result, it would be expected that any difference attributed to a larger sample size alone would have minimal clinical relevance.

## 5 Conclusion and recommendation

In light of the findings of the present results, a rehabilitation program for both the operated and non-operated limbs is necessary for patients who have had complete hip replacements to establish bilateral hip joint stability. This maintains both hips’ symmetrical lateral stability while they walk. Additionally, both ASIS should be kept in their normal horizontal alignment to avoid substituting their trunk when walking, which might cause the vertebral column to acquire an aberrant curve and put more strain on the operated hip. Consequently, the early failure of the prosthetic hip is attributable to increased stress on the operated hip. Given the current study’s findings, we also suggested, as a preventive strategy, that all of the anti-gravity muscles in the hips and back be rehabilitated.

Furthermore, particular emphasis was devoted to the female-operated hip, which saw a higher and considerable rise in hip abduction angle during walking, potentially exposing the hip to recurrent dislocation after surgery. As a result, the physiotherapy rehabilitation program concentrated on the female group closest to their abductors. The relevance of abductors increases, particularly during mid-stance, when the body weight is concentrated on one leg with a higher GRF value, causing it to be directed more medially to the operating hip and necessitating bigger hip abductor moments. Furthermore, acquiring lateral hip stability directly affects the typical lateral stability mechanics of the knee and ankle joints during walking.

## Data Availability

The original contributions presented in the study are included in the article/Supplementary Material; further inquiries can be directed to the corresponding author.
